# The potential of the Medical Digital Twin in diabetes management: a review

**DOI:** 10.3389/fmed.2023.1178912

**Published:** 2023-07-20

**Authors:** Yanting Chu, Shanhong Li, Jie Tang, Huiqun Wu

**Affiliations:** Department of Medical Informatics, Medical School of Nantong University, Nantong, China

**Keywords:** digital twin, diabetes management, diabetes mellitus, artificial intelligence, virtual reality

## Abstract

Diabetes is a chronic prevalent disease that must be managed to improve the patient's quality of life. However, the limited healthcare management resources compared to the large diabetes mellitus (DM) population are an obstacle that needs modern information technology to improve. Digital twin (DT) is a relatively new approach that has emerged as a viable tool in several sectors of healthcare, and there have been some publications on DT in disease management. The systematic summary of the use of DTs and its potential applications in DM is less reported. In this review, we summarized the key techniques of DTs, proposed the potentials of DTs in DM management from different aspects, and discussed the concerns of this novel technique in DM management.

## 1. Introduction

In March 2011, the United States Air Force Research Laboratory (AFRL) presented a seminal discourse titled “Condition-based Maintenance Plus Structural Integrity (CBM + SI) and the Airframe Digital Twin.” This marked the inaugural explicit mention of the “digital twin” (DT) concept. The AFRL further elucidated a conceptual model of DT technology, specifically for predicting the structural lifespan of aircraft in the same year (1). The National Aeronautics and Space Administration (NASA) defines a “DT” as a comprehensive utilization of physical models, sensors, operational history, and other relevant data to amalgamate the entire process of multi-disciplinary, multi-physical, multi-scale, and multi-probability simulation. This process culminates in a mapping within the virtual space that mirrors the entire life cycle of the corresponding physical equipment. In recent years, the DT concept has progressively permeated the medical field. For instance, in the realm of cardiovascular systems, extant technology has advanced to the point where it is feasible to construct fully personalized, high-resolution models of the entire heart (2). The VirtaMed virtual surgical training system LaparoS has been a trailblazer in applying DT technology to replicate intricate surgical scenarios. Similarly, the University of Linkoping in Sweden has embarked on an innovative project aimed at investigating the potential of DTs in various aspects of medical practice, including medical education, heart diagnosis, and medical implant planning (3).

The evolution of health services in contemporary society is evident, with a shift toward a more technologically advanced approach. The integration of artificial intelligence (AI) and point-of-care sensors has revolutionized traditional face-to-face disease management, transitioning it to an online platform. AI, when synergized with DTs, can construct models that enhance the efficiency, precision, and promptness of patient care. In the realm of healthcare, DT offers a unique perspective on self-quantification, potentially establishing a new paradigm for disease management (4). Since 2017, Gartner's emerging technology maturity curve has predicted that DT could reach a level of mature application within the next 5–10 years. Presently, the innovative applications of DT are transforming the manufacturing industry. DT has facilitated the integration of digital and physical spaces throughout the entire lifecycle of spacecraft (5). Furthermore, the use of DT in collaborative painting robots has improved worker safety and health (6). Despite these advancements, the application of DT in the medical sector remains limited. It is reported that 47% of DT applications are in the smart cities and urban spaces sector and 17% of DT applications are in the manufacturing sector, but only a meager 1% of DT applications are in the medical sector (7). Specifically, the application of DT in managing chronic diseases, such as diabetes mellitus (DM), is significantly lagging. In light of this, we have reviewed the literature on DT, summarizing the key techniques and exploring the potential applications of DT in DM management. Our goal is to anticipate future trends and directions in this field.

## 2. The key techniques with regard to MeDigiT in DM management

**Figure 1:**
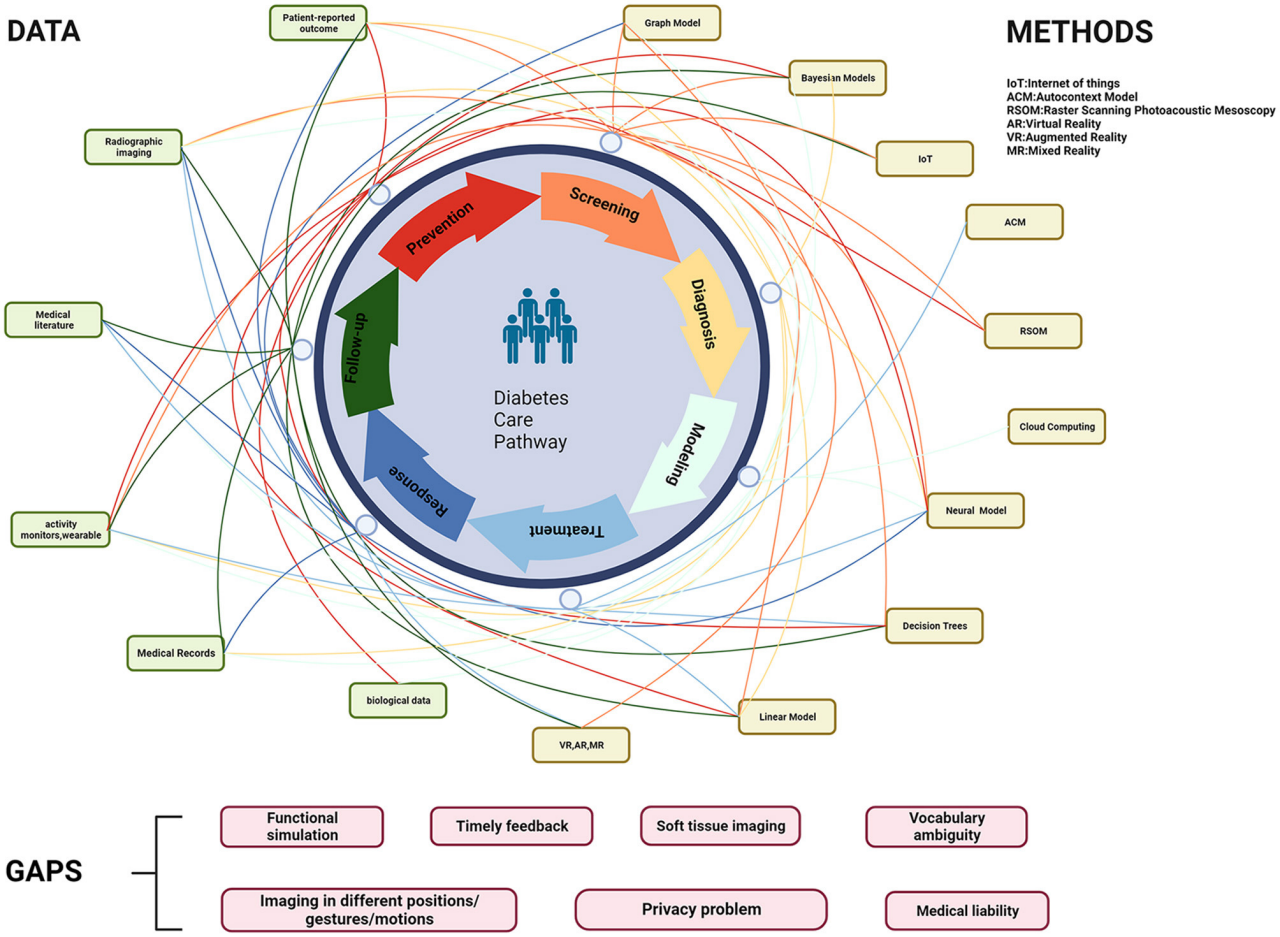
Involved data and methods in the DT cycle of the diabetes care pathway. IoT, Internet of Things; ACM, autocontext model; RSOM, raster scanning photoacoustic mesoscopy; VR, virtual reality; AR, augmented reality; MR, mixed reality.

The concept of a Medical Digital Twin (MeDigiT) can be defined as a system that amalgamates various data science methodologies, each tailored to predict specific aspects of a patient's health (8). As depicted in Figure 1, the DT cycle in the diabetes care pathway incorporates a multitude of techniques at every stage of the diabetes mellitus (DM) management cycle. This includes pre-disease management, disease management, and post-disease management. The integration of diverse data sources and the application of various methods for data collection, modeling, and visualization is fundamental to the efficacy of DT. In the pre-disease management phase, DT can assess an individual's risk of developing DM by analyzing contributing factors such as obesity, a sedentary lifestyle, and genetic predisposition. This allows for the provision of preemptive interventions to prevent the onset of DM. For patients already diagnosed with DM, their DT counterpart can be utilized by healthcare professionals to administer personalized treatment options. For instance, any abnormality in blood sugar levels detected by real-time glucose monitors can be relayed to the DT system, which can then adjust the insulin dosage accordingly. In terms of post-disease management, DTs have the potential to predict diabetic complications such as cardiovascular disease, kidney failure, and vision loss during follow-up sessions. However, despite the promising advancements in technology, there remain challenges in achieving an optimal DT system. For instance, vocabulary ambiguity in electronic medical records (EMRs) is a common issue. Variations in body imaging, particularly in soft tissue imaging due to different patient positions, gestures, or motions, can lead to inconsistencies in the anatomical DT. The functional simulation methods for physical DT are still complex and time-consuming, which hampers timely feedback. Additionally, concerns regarding patient data privacy and the lack of medical liability in decision-making by DT persist. These challenges underscore the need for further research and development in the field of DT (Figure 1).

**Figure 2:**
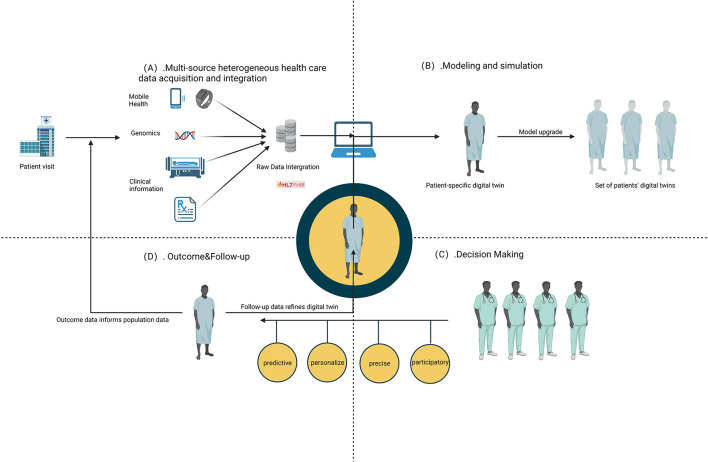
Key techniques involved in DT. **(A)** Multi-source heterogeneous health care data acquisition and integration. **(B)** Modeling and simulation. **(C)** Outcome and follow-up. **(D)** Decision making.

As depicted in Figure 2, the initial step in managing DM involves the crucial task of collecting and integrating multiple variables related to the disease. Subsequently, the MeDigiT system integrates these biomedical data for advanced modeling and simulation. For instance, 3D modeling can be executed using imaging data, while time series modeling can be constructed using data from wearable devices, among other techniques. Following the diagnosis of DM, clinicians administer a drug or treatment plan based on the decision-making support provided by the DT system. Finally, the most effective treatment or medication is delivered to the patient. In this context, we have reviewed the literature and summarized the potential key techniques involved in this process as follows.

### 2.1. The acquisition and integration of multi-source heterogeneous healthcare data

The application of DT in healthcare begins with the acquisition of multi-source, heterogeneous patient data. The evolution of sensor technology has made it possible to consolidate data from wearables, medical imaging, and EMR onto a single platform. This comprehensive data integration enables medical professionals to deliver more efficient patient care. In the realm of data collection, MeDigiT can digitize and quantify an individual's data at various levels. High-throughput sequencing data and expression profiles can be obtained from T2DM patients' biological samples using multiple omics techniques, such as genomics, transcriptomics, proteomics, and epigenomics (9–11). The advancement and optimization of medical imaging techniques have also provided a reliable data source for MeDigiT. For instance, Philips has proposed HeartModel, a system that simulates every frame of the cardiac cycle, thereby providing critical information for clinical strategy (12). Continuous bio-signals, collected through wearable devices, are crucial for health monitoring. Microsoft has proposed an Internet of Things (IoT) application platform that seamlessly connects various medical wearables to IoT central instances. This platform enables the monitoring and management of devices by customizing rules to specific device data and triggering corresponding alerts. Moreover, lifestyle data (such as diet, smoking, drinking, and drug use), environmental data (including living and working conditions), EMR, mobile apps, social media, and wearables all contribute to the data pool for MeDigiT. Given the complexity, diversity, and scale of these data, effective algorithms are required to integrate these multi-source data streams.

### 2.2. Digital modeling and simulation

The MeDigiT system, established from patient data collected through sensors, medical treatment facilities, EMRs, and more, can be utilized to tailor interventions and therapies and monitor patient responses. For example, Pfizer has used AnyLogic software to assist physicians in modeling and analyzing optimized drug dosages for peripheral neuropathy (13). Digital modeling and simulation techniques are crucial tools for accurately reflecting and modeling a patient's condition. For instance, anatomical models of the body and internal organs, created using 3D modeling tools, such as Mimics, Simpleware, and 3dSlicer (14), are invaluable for clinicians making diagnoses. Physical models, such as biomechanical models for blood vessels, muscles, and bones, provide doctors with the physical mechanisms needed to calculate and estimate full-cycle and full-field dynamic simulations. Virtual reality (VR) simulation systems have also been developed for personalized interventions, such as percutaneous coronary intervention (PCI). These systems combine the patient's cardiac dynamic model to bridge the gap between the physical world and the virtual scene (15). Ilyan et al. (16) simulated the coronary vascular system of the human heart to assist in the treatment of heart disease. MeDigiT can also develop physiological models using signals from the IoT. For instance, in traditional cardiac radiofrequency ablation surgery, doctors have had to 'imagine' all suspected heart conditions due to technical limitations. They would then rule out some of these conditions by applying electrical stimulation to the patient's heart to get an accurate diagnosis. However, Wu et al. (17) developed a model-based cardiac radiofrequency ablation procedure assist system, which used DT to enumerate suspected cardiac conditions in the diagnostic process through cardiac electrophysiological models. Beyond these models, biochemical models can simulate functions of various systems, such as the endocrine system or liver. Simulation is fundamental for further model-based analysis, training, and prediction. Digital modeling and simulation are often used in tandem, with simulations being performed on the model.

### 2.3. Decision-making and AI

The full potential of MeDigiT cannot be realized without the integration of AI. As stated by Robert Hayward, “Clinical Decision Support Systems (CDSS) link health observations with health knowledge to influence health choices by clinicians for improved healthcare.” CDSS are computerized systems that pair patient clinical information with a knowledge base, aiding medical staff in disease diagnosis and the optimization of treatment plans. By prompting interventions in the diagnosis and treatment process, CDSS can reduce medical errors and enhance the quality of care. AI technology enables MeDigiT to achieve its decision-making objectives, which include description, diagnosis, and prediction. There are two primary methods for achieving decision-making. The first is through an expert knowledge base, which mirrors how doctors make decisions based on their experience. The system extracts decision rules and analyzes patient data as variables to assess the patient's situation and draw conclusions. This approach allows the computer to perform enumeration and reasoning tasks and present the results in a form that aligns with the physician's expertise. Consequently, physicians can make accurate decisions with the system's assistance, using explanatory reasoning rules. The second method of decision-making involves machine learning, including the latest deep learning (DL) techniques. For instance, Zhang et al. (18) developed a deep neural network model that captures the relationship between contexts through risk code keywords. An analytical method capable of generating treatment pathways was developed and validated (19) with 27,904 diabetic patients. While these methods can effectively assist doctors in decision-making, medical decisions are complex and often fraught with uncertainty. When decisions involve moral and ethical issues, it can be challenging for doctors to explain their reasoning. In response to this, behavioral artificial intelligence technology (BAIT) (20) has been proposed. BAIT can predict the probability of a patient's condition, thereby assisting doctors in making decisions within specific contexts.

### 2.4. Feedback and control

The integration of patient data, collected by a multitude of sensors, with AI and IoT technologies, can facilitate the creation of MeDigiT. This digital twin can simulate predicted outcomes and assist doctors in making clinical decisions, enabling treatment feedback to be relayed back to the patient in the physical world. As treatment is administered, the MeDigiT model can be updated in real time to reflect the physiological changes in patients. This feedback mechanism allows the DT to establish a closed-loop interaction between the real and virtual worlds. A controlled trial in South Korea demonstrated the effectiveness of this approach in managing diabetic patients using cell phone glucose monitoring and feedback systems. The study showed that patients who received feedback had more effective glycemic control (21). Electronic biofeedback therapy, which uses modern electronic instruments to convert bioelectricity into sound, light, and other signals, has been used for patient rehabilitation. Song et al. (22) verified its MeDigiT with a man–machine–environment fusion to realize dynamic human–machine interaction. In addition to its crucial role in clinical treatment, feedback can also be used in the context of virtual candidate drugs applied to the DT. The Swedish Digital Twin Consortium (SDTC) proposed a strategy for this purpose: first, constructing unlimited copies of network models of all molecular, phenotypic, and environmental factors relevant to disease mechanisms in individual patients, and second, computationally treating those DTs with 1,000's of drugs to identify the best ones. Eventually, drugs selected by DTs are used to treat real-world patients as feedback (23). Alacris is an example of a MeDigiT that virtually tests which drugs can stop cancer cells. The results suggested that everolimus, usually approved for the treatment of breast and kidney cancer, seemed to work in the case of mucosal tumors insensitive to chemotherapy, immunotherapy, and radiotherapy, reducing the rate of dividing cells to 15% (24). Furthermore, to improve patient adherence and accessibility to chronic disease management, digital therapeutics (DTx) have emerged as an innovative approach to transcend the limitations of traditional pharmacotherapy. DTx, a software program-driven, evidence-based intervention program, can treat, manage, or control disease. It can be conducted alone or in conjunction with drugs, medical devices, or other therapies. DTx can digitally translate existing medical principles, medical guidelines, or standard treatment protocols into application-driven interventions. In summary, MeDigiT assists physicians in leveraging big data and expanding the value of that data to make clinical decisions by providing better feedback to the patient on the course of their illness.

## 3. The application of MeDigiT in terms of DM management at multi-scale levels

**Figure 3:**
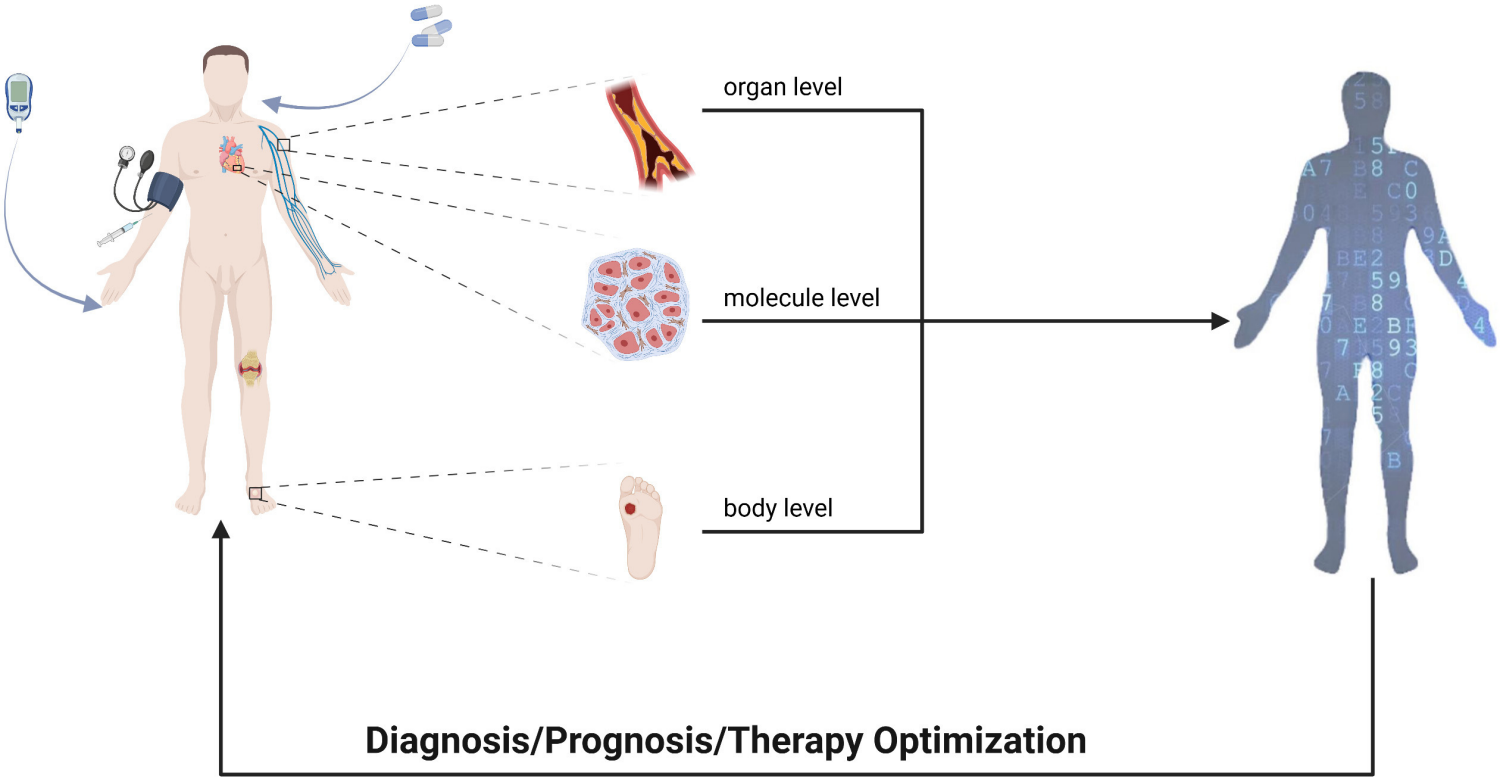
Diagram of applications of DT in diabetic patients at different scales.

Diabetes mellitus is a complex disease with a multitude of contributing factors, including genetic predispositions, immune disorders, microbial infections and their toxins, free radical toxins, and psychological factors. These factors can lead to metabolic disorders such as hypoglycemia and insulin resistance, resulting in complications such as the kidney, eye, and foot failure. MeDigiT, a dynamic digital replica of the patient, can assist healthcare professionals in understanding a patient's medical status and providing personalized care for DM patients. MeDigiT offers an effective blueprint for the treatment of chronic diseases such as DM. For diabetic patients, complications from DM are a major cause of disability or death, making the management of DM of utmost importance. Studies have shown significant improvements in self-care for patients with multiple myeloma when they received a combination of online and offline health education, allowing for continuous disease care and prevention of complications (24). MeDigiT has potential in this whole disease management cycle, serving as a core component at multi-scale levels for diabetic patients (Figure 3).

### 3.1. The body level

MeDigiT plays a crucial role in DM management through diet, exercise, and insulin function. Thamotharan et al. (25) proposed a human digital twin (HDT) framework and IoT architecture for personalized management of T2DM in older adults. The framework combines deep learning (DL) models and mathematical models based on various patient data and can personalize insulin administration according to the patient's different statuses. Deployment and testing have proven that HDT is effective in personalized management and treatment of DM. Similarly, Twin Health proposed the whole-body DT for DM by monitoring patient sensor data and offering personalized recommendations (26). Shamanna et al. (27, 28) piloted a twin precision treatment (TPT) program in patients with diabetic hypertension to achieve improvements in insulin resistance and hypertension. The program used the Twin mobile app, continuous glucose monitors (CGM), Fitbit Charge 2 sensor watch, digital Bluetooth-enabled blood pressure meter (TAIDOC TD-3140), and Powermax BCA-130 Bluetooth Smart Scale to obtain and analyze physiological data from DM patients. It created a MeDigiT that dynamically represents the metabolic status of the DM patient and provides personalized interventions. Currently, a “metabolic DT” (MDTwin) (29) personalizes insulin dosing and delivery patterns to patients by assessing individual glycemic responses to a high-fat, high-protein diet to achieve optimal patient blood glucose levels. In addition, family diet and various educational and environmental data will be recorded, and a DT around the person can be constructed if necessary. MeDigiT can use various data and information to assess the condition of the human body and give a personalized assessment. With the help of expert knowledge systems, health assessments and disease diagnoses are made automatically, and the information is fed back to the patient to make improvements accordingly. Through the MeDigiT of a diabetic patient, the doctor can monitor the patient's condition and enable accurate and timely DM management. The Cleveland Clinic (30) conducted a pilot randomized controlled trial (RCT) of DT precision treatment which provides precise management of nutrition, activity, and sleep with trained health coaches through the app and via telephone to ensure that the average blood glucose of the day will be consistently maintained within the optimal range. In the RCT, the DT platform was used to obtain personalized multidimensional data of patients, and the TPT treatment system was integrated and predicted to give reasonable dietary guidance. Therefore, the results are expected to provide a basis for the application of MeDigiT in the treatment and management of DM at the patient level.

### 3.2. The organ level

Diabetes mellitus is often associated with several chronic complications, including retinopathy, atherosclerotic disease, and diabetic foot. In addition to constructing a comprehensive MeDigiT for DM management, it is also feasible to create individual organ twins to prevent and treat DM complications. Traditionally, pathological changes at the organ level are detected and assessed through medical imaging. However, with the advent of MeDigiT, digital replicas can be developed to model and simulate the corresponding diseased organs. These patient-specific models are crucial for planning and selecting the appropriate intervention. For instance, Orcajo et al. proposed a foot twin, which can be utilized for improved diagnosis, tailored treatments, and intervention risk reduction to achieve the best therapy after testing new surgical procedures (31). Furthermore, the introduction of MeDigiT can assist clinicians in developing optimized therapy strategies for patients with chronic heart failure (32). The Living Heart Project, developed by Dassault Systèmes, a French software company, considers all aspects of cardiac function (e.g., blood flow, mechanics, and electrical pulses) to aid clinicians in predicting patient outcomes. Siemens has also developed a cardiac DT model that is expected to simulate a patient's heart, including size, ejection fraction, and muscle contraction. The DT of a patient's heart allows for real-time monitoring of the patient's heart, and by monitoring the patient's heart function data and providing timely feedback to the physician, it also offers the patient a timely prognosis as well as precise treatment.

### 3.3. The molecular level

A person's biogenetic characteristics can be directly inherited from their grandparents. Concurrently, the sequenced genetic data of an individual and their parents will be recorded in real time, and every medical examination conducted by a healthcare institution will be transmitted to the network with the consent of the individual. Cells, as the basic structural and functional units of the human body, store genetic information. With the advancement of life observation technology to the single-cell level, MeDigiT can also be applied to the segmentation, detection, and tracking of stem cell images, with its accuracy and recall in stem cell image segmentation found to be superior to those of phase difference (33). Li et al. (34) constructed a MeDigiT framework based on dynamic single cells, which can prioritize the upstream regulator (UR) gene of biomarkers and drug discovery according to the dynamic changes of MeDigiT in seasonal allergic rhinitis. Chen et al. (35) proposed an innovative virtual cell experiment scheme to establish the human Ensemble Cell Atlas (hECA) system, a cell-centered human cell graph. The hECA can be used to conduct drug experiments on virtual human cells, thereby improving drug development efficiency and reducing the cost of human clinical trials. Type 1 DM and advanced T2DM are due to pancreatic β-cell loss and failure, leading to inadequate insulin production. Some studies have identified molecular biomarkers for the diagnosis of DM status, with the potential to detect DM (36, 37). The EU combining photoacoustic imaging phenotyping and multiomics to advance diabetes care (OPTOMICS) validated a MeDigiT model of the static and dynamic processes involved in the development of T2DM, by combining molecular biomarkers and a non-invasive phenotyping technique called raster scanning optoacoustic mesoscopy (38). This MeDigiT model, combined with an in-depth molecular phenotype of the individual at the DNA, protein, and metabolite levels, offers advantages for the prediction and early detection of diseased individuals, thereby improving the overall likelihood of prevention. The ILET bionic pancreas is a new type of insulin delivery system, which can achieve the maximum serum drug concentration in a short time by injecting insulin with continuous glucose monitoring (39). In summary, the clinical efficacy, benefit, and cost-effectiveness of MeDigiT in the treatment of DM at the molecular level are considerable. This approach holds significant potential to aid in the treatment and research of DM in the future.

## 4. The limitations and concerns of MeDigiT in DM management

### 4.1. Interoperability of healthcare data

The realization of MeDigiT is contingent upon the aggregation and integration of multiple sources of data, which in turn relies on solutions for interoperability. In the integration of large-scale biomedical data, standardization emerges as a significant means of integrating and standardizing multi-source heterogeneous data in non-standard formats to facilitate subsequent utilization and analysis. The Health Level Seven (HL7) standard has been released to access patient data (40) in an interoperable manner. This standard enables the access and exchange of integrated and collaborative use of data in a coordinated manner between different information systems, devices, applications, and programs, within and across institutional, regional, and national boundaries. For instance, an AI model predicts the 5-year risk of end-stage renal disease in T2DM using data from the EMR, enabling preprocessing of unstructured data in compliance with the HL7 standard (41).

### 4.2. Privacy and security challenges

The implementation of MeDigiT presents a significant challenge when it comes to privacy violations, which involve social and ethical risks (42, 43). Achieving interoperability implies the possibility of multi-platform connectivity and information transfer. However, data sharing requires a strict ethical review to protect patient privacy. Foreign attacks could potentially destroy the program code and cause harm to the patient's interests or even life. Healthcare providers or any other organization possessing a persistent, detailed picture of a person's biological, genetic, physical, and lifestyle information over time poses a significant privacy risk. Therefore, it is crucial to obtain absolute consent from stakeholders when dealing with these data. Hackers could potentially steal gene data from gene banks to carry out criminal activities. Assuming that DNA can be obtained through cybercrime, there is a risk that criminals will place DNA samples at the crime scene. Therefore, for security issues including medical data security, user platform security, and network security, it is necessary to establish a security management system. Furthermore, running such highly secure and sensitive software must be immune to disruption and loss of medical data access when software patches are upgraded. For privacy issues, various technologies can be used for encryption, such as passwords, fingerprints, and iris recognition. Access to or modification of the MeDigiT should be granted only to a physician or professional with the appropriate permissions.

### 4.3. Ethical and socioeconomic considerations

The implementation of MeDigiT can amplify the individual differences that already exist among humans, including health, longevity, and strength. For instance, an athlete's performance enhanced through long-term training, diet, and lifestyle may be comparable to that of a person who has improved through the use of DT-based drugs. This poses an inequality problem in competitive arenas. When this inequality is extrapolated to society at large, one can access high-volume data related to someone's genes, metabolism, lifestyle, etc. For example, if MeDigiT predicts that an individual is likely to suffer from a certain disease, it may facilitate healthcare, but this result will become part of the person's identity and could eventually influence society's judgment of the person, labeling them as “sick”. Furthermore, MeDigiT can also exacerbate pre-existing socioeconomic gaps. People who can afford to pay for the services can gain knowledge that others may not have by testing treatment with MeDigiT. The countries that are relatively wealthy and possess MeDigiT research and development facilities and the resulting intellectual property can widen the gap between rich and poor nations. If the development and design of the DT itself are biased (race and gender), then patients may be treated discriminatively (44).

## 5. Conclusion

In conclusion, DT is an emerging technology based on data, with the model as the core, and software as the carrier, by describing the physical world, diagnosing the physical world, and then gradually upgrading to predicting the physical world and finally making decisions. In healthcare, MeDigiT has the advantage of lowering costs, reducing animal testing, and improving disease prevention. It will continue to grow in popularity and accelerate its current trend with the development of AI and computer technology. To reach the goal of fully applying the MeDigiT to DM management, there is still a need to continue to advance its exploration in data integration, modeling and simulation, and decision-making, as well as feedback and control.

## Author contributions

YC and HW contributed to conception and design of the study and completed the critical review and final approval. YC wrote the first draft of the manuscript. JT, HW, and SL did the logistical support. All authors contributed to manuscript revision, read, and approved the submitted version.
